# Prakash Sarvotham Shetty (1943–2018)

**DOI:** 10.1017/jns.2018.20

**Published:** 2018-11-06

**Authors:** Paul Trayhurn, W. Philip T. James

**Affiliations:** 1 Emeritus Professor of Nutritional Biology, University of Liverpool, Liverpool, UK; 2 Emeritus Professor, Clore Laboratory, University of Buckingham, Hunter Street, Buckingham MK18 1EG, UK; 3 Honorary Professor of Nutrition, London School of Hygiene and Tropical Medicine, Keppel Street, London WC1E 7HT, UK

**Figure:**
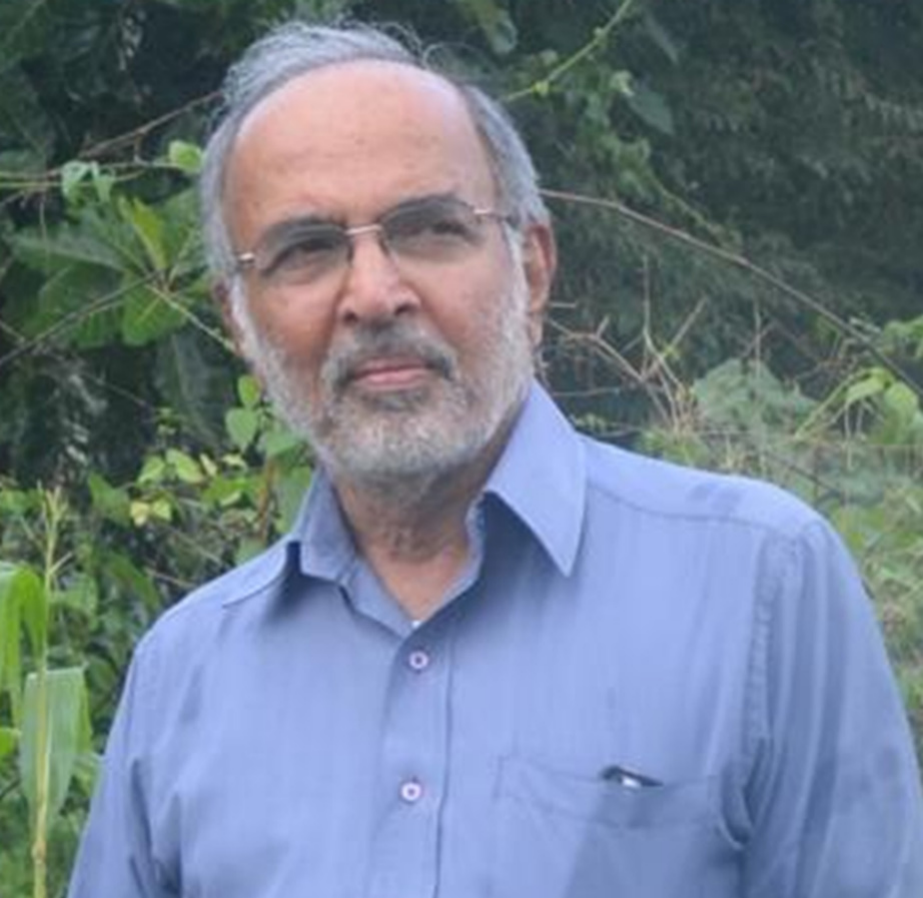
Prakash Shetty. Reproduced by courtesy of LANSA.

Our great friend and colleague Prakash Shetty passed away in London on 3 September 2018 after suffering with cancer. He was a highly distinguished nutritionist whose career divided into periods in India, and then in the UK and Italy. Although he was based in Europe for the last 25 years, he always maintained strong links with the nutrition community in India and remained an Emeritus Professor at St John's Medical College in Bengaluru (formerly Bangalore).

Prakash was born in India on 28 September 1943. He trained in medicine at the prestigious Christian Medical College in Vellore, a Christian Medical Institution which this year celebrates its centenary in medical education. He was a star student at Vellore and after graduation became a tutor in the college and took his MD research degree there in 1972 on the effect of carbonic anhydrase inhibition on gastric secretion. He then took up that same year a post as an Assistant Professor in the Physiology Department at St John's Medical College, Bengaluru, St John's being another of the leading medical colleges in India. In 1977 he came to the UK to undertake his PhD under the supervision of one of us (WPTJ) at the MRC Dunn Clinical Nutrition Centre in Cambridge, beginning his long-term research interest in the physiological consequences of protein and energy restriction in humans. He had written some 2–3 years earlier while WPTJ was at the London School of Hygiene and Tropical Medicine (LSHTM) enquiring about the possibility of undertaking a PhD in the UK, and was told that he needed to win a Commonwealth Scholarship – which he then successfully achieved.

Prakash settled into Cambridge life with remarkable rapidity and quickly became the person who was able to cope with the most obstructive, angular, or timid of colleagues. He was instrumental in helping WPTJ to progress Dunn Clinical's research by taking over a derelict hospital ward to facilitate clinical metabolic studies – for which the group was subsequently applauded by the UK Medical Research Council. His research for his PhD focused on the metabolic responses to dieting and semi-starvation with a particular interest in the sympathetic system and its potential role in controlling differential brown adipose tissue activity in lean and familial obese adults. His productivity was remarkable, and he played a key role in our studies on thermogenesis in obesity, which was a major part of the programme of the Energy Group in Cambridge at that time.

Not only was Prakash an excellent clinical scientist, but he also became an exceptionally good friend to both of us – and a friend to other colleagues and fellow students. He often came to WPTJ’s house in Balsham outside Cambridge where he helped entertain a variety of foreign guests as well as more prosaically enthusiastically participating in home maintenance tasks such as cleaning out the garden pond – delighting in extracting newts and frogs from the sludge! He was also a regular visitor to PT’s home, again becoming a close family friend, and so much so that he became a godfather to the Trayhurns’ eldest son. Some 30 years later he, and his wife Nandini, were able to witness his Cambridge godson's wedding.

After Cambridge, Prakash returned to St John's and was rapidly promoted to Associate Professor, and then Full Professor and Director of the Nutrition Research Centre. His research at St John's was shaped by the interests and expertise that he had gained at Dunn Clinical. He worked extensively on energy metabolism, particularly energy expenditure and the impact of the autonomic nervous system and the responses to undernutrition; he also developed interests in body composition. In his early years at St John's he additionally worked on dietary fibre and transit time in association with his student Anura Kurpad – who went on to succeed him as Head of Physiology at St John's. He was responsible for the considerable achievement of developing the very first whole-body calorimeter in Asia, receiving funding from the Wellcome Trust in the UK.

Prakash's success at St John's, together with his increasing international reputation, led to his being asked to apply for the Chair of Nutrition at the LSHTM which had been vacant since the retirement of Professor John Waterlow. He moved to the London School in 1993, but spent the preceding months at the Rowett Research Institute in Aberdeen where we were both then based. He renewed his scientific collaboration with WPTJ in Aberdeen, setting out the importance of assessing BMI when evaluating not only obesity but undernutrition and energy needs as well. This was important in an FAO (UN Food and Agriculture Organization) context where all studies on global hunger were starting to be based on the research and analyses undertaken at the Dunn and then at the Rowett. He came to the UK with his wife Nandini, a medical microbiologist, and their two children, Dushyant and Meghna. The period in Aberdeen provided an opportunity for the family as a whole to adapt to life in the UK, which while at first challenging has led to considerable success for each of them in their chosen careers.

At the LSHTM, Prakash reformed the School's nutrition research with a new focus on public health and he became Head of the Public Health Nutrition Unit. As part of the change in emphasis the well-established nutrition course was renamed the MSc in Public Health Nutrition and Prakash published his book *Nutrition, Immunity and Infection*, illustrating his switch to a public health focus. In 2001 he left the LSHTM to take on the challenging position of Chief of Nutrition Planning, Assessment and Evaluation at the FAO in Rome. This involved an extraordinarily wide range of scientific and political issues, which he handled with his usual skill, until retirement in 2005 – at the early age of 62 years as the UN system required. However, he immediately became a Visiting Professor in Public Health Nutrition at the University of Southampton, a position he held until 2015, contributing his considerable flair in teaching. In Southampton, as at St John's and the LSHTM, he was highly regarded as a teacher, and one who was extremely supportive of his students. From 2006 to 2011, he also took on a substantial editorial responsibility as Editor-in-Chief of the *European Journal of Clinical Nutrition*, leading its continued development as a major international nutrition forum.

Prakash's final formal role was as CEO of the Research Programme Consortium of ‘Leveraging Agriculture for Nutrition in South Asia’ (LANSA) led by the M. S. Swaminathan Research Foundation (MSSRF) and supported by the UK Department for International Development. His colleagues at LANSA have commented on his leadership and eminence, both as an intellectual and as an administrator who ‘was always ready to provide guidance, offer advice and extend support as required’. Here again he showed his personal capacity to solve problems and difficult issues, and then to lead a progressive collaborative effort across national boundaries. This usually involved a series of personal phone calls rather than negotiating as normally done through formal letters, etc.

Prakash had very wide intellectual interests, reading far beyond the boundaries of nutrition and of science. When he first arrived in the UK, WPTJ had to attend a meeting in Brussels and so arranged to meet him at King's Cross Station in London. In that first exchange, Prakash asked whether it would be acceptable for him to present a special lecture to the Royal Geographical Society in London on the historical significance of sculptures with a medical theme in Hindu temples in India. It was slowly extracted from him that he was at the time the leading authority on the subject. This was for us the first of many such indications of the considerable depth and range of his scholarship. Others include his extensive knowledge of literature, frequently exchanging information on new authors with PT, introducing him for example to the thrillers of the Icelandic writer Arnaldur Indriðason well before he was known in the English-speaking world. This particular example reflects how much Prakash was a citizen of the world – indeed, the world had no real boundaries for him. Nevertheless, it was always recognised that philosophically he was strongly, and rightly, rooted in his background and upbringing in India.

We are both very conscious of his personal generosity. WPTJ visited him several times while he was in Bengaluru and witnessed the way in which his career post-Cambridge took off in the most gratifying manner. On one occasion, WPTJ had asked if his mother could accompany him to Bengaluru as she had never been to India. Prakash was at once incredibly welcoming and organised a special programme for her while the collaborative scientific work was being pursued. He arranged for the James family to stay in his own home and there was considerable shock – and embarrassment – when it was discovered that on the day before arrival his wife had been admitted to hospital to deliver their first child. PT recalls that while he had not been able to visit him in Bengaluru, the trip that was arranged having to be cancelled, Prakash had often sent generous gifts reflecting Indian culture to members of the family – a sari, traditional children's clothes and LPs of ragas of classical Indian music. Many others will have their own examples of his kindness and generosity.

One of Prakash's very special qualities was the facility with which he adapted to different environments – he always seemed to embrace, and be embraced, wherever he was. Whether in Bengaluru, Cambridge, Aberdeen, London or Rome, his sensitivity, warmth, humour and sheer goodwill was a constant – and what always shone through was a remarkable person with enormous empathy and understanding. We will greatly miss our splendid friend.

Prakash is survived by his wife Nandini, a highly regarded medical microbiologist at Public Health England, his son Dushyant, a consultant gastrointestinal interventional radiologist, his daughter Meghna, a lawyer with an international law firm, and his two grandsons.

